# Membrane-Anchored Hairless Protein Restrains Notch Signaling Activity

**DOI:** 10.3390/genes11111315

**Published:** 2020-11-06

**Authors:** Dieter Maier

**Affiliations:** Deptartment of General Genetics 190g, University of Hohenheim, Garbenstr. 30, 70599 Stuttgart, Germany; dieter.maier@uni-hohenheim.de

**Keywords:** notch signaling, suppressor of hairless, Hairless, *Drosophila*, membrane-anchor, sequestration, repressor complex, transcriptional regulation

## Abstract

The Notch signaling pathway governs cell-to-cell communication in higher eukaryotes. In *Drosophila*, after cleavage of the transmembrane receptor Notch, the intracellular domain of Notch (ICN) binds to the transducer Suppressor of Hairless (Su(H)) and shuttles into the nucleus to activate Notch target genes. Similarly, the Notch antagonist Hairless transfers Su(H) into the nucleus to repress Notch target genes. With the aim to prevent Su(H) nuclear translocation, Hairless was fused to a transmembrane domain to anchor the protein at membranes. Indeed, endogenous Su(H) co-localized with membrane-anchored Hairless, demonstrating their binding in the cytoplasm. Moreover, adult phenotypes uncovered a loss of Notch activity, in support of membrane-anchored Hairless sequestering Su(H) in the cytosol. A combined overexpression of membrane-anchored Hairless with Su(H) lead to tissue proliferation, which is in contrast to the observed apoptosis after ectopic co-overexpression of the wild-type genes, indicating a shift to a gain of Notch activity. A mixed response, general de-repression of Notch signaling output, plus inhibition at places of highest Notch activity, perhaps reflects Su(H)’s role as activator and repressor, supported by results obtained with the Hairless-binding deficient Su(H)^LLL^ mutant, inducing activation only. Overall, the results strengthen the idea of Su(H) and Hairless complex formation within the cytosolic compartment.

## 1. Introduction

The development of eukaryotic animals depends on a multitude of developmental decisions underlying cellular differentiation, triggered by several signaling pathways. One example is the Notch signaling pathway that allows communication amongst neighboring cells. Name-giving to this pathway is the receptor Notch, a large transmembrane protein, which is activated by binding to its transmembrane ligands, named Delta and Serrate in *Drosophila*, that are presented on the adjacent cell. Consequently to the activation, the intracellular domain of Notch (ICN) is cleaved and released into the cytosol of the responding cell [1]. Intriguingly, ICN itself plays an important role in signal transduction. Firstly, it is involved in the nuclear translocation of the transcription factor Suppressor of Hairless (Su(H)) whereby binding occurs in the cytosol [2,3,4]. Secondly, it acts as a transcriptional coactivator of Su(H) in conjunction with other co-factors in an activator complex [5,6,7,8,9]. Indeed, activator complex formation is highly conserved throughout the animal kingdom [10,11,12].

Likewise, *Drosophila* Su(H) is extremely well-conserved in structure and function with its vertebrate and invertebrate orthologues of the CSL family [13,14,15]. CSL is the abbreviation for human CBF1 (C-promoter Binding Factor 1, also named RBPJ for recombination signal-binding protein for immunoglobulin kappa J region), *Drosophila* Su(H), and the nematode *Caenorhabditis elegans* protein Lag1 (abnormal cell LINeage-12 (Lin-12) and abnormal germ-line proliferation phenotype-1 (Glp-1)). For example, murine RBPJ is 80% identical to *Drosophila* Su(H) at the amino acid level [14]. In fact, RBPJ can even largely replace Su(H) activity in the fly [16,17]. CSL transcription factors all share three functional domains: the N-terminal domain (NTD) and the β trefoil domain (BTD) contact the DNA, whereas the BTD and the C-terminal domain (CTD) contact ICN [8,18,19,20]. CSL proteins in general act as a molecular switch, since they not only serve as transcriptional activators of Notch target genes, but also as repressors in the absence of signal. In this case, CSL assembles repressor complexes on Notch target gene promoters [5,21]. However, repression mechanisms are more diverged: in *Drosophila*, the central player is Hairless (H), which functions as an adaptor in the repressor complex, binding Su(H) as well as the general co-repressors Groucho and C-terminal binding protein, eventually resulting in gene silencing [22,23,24,25].

Hairless binds with nanomolar affinity to the C-terminal domain of Su(H) [26,27]. The crystal structure of the repressor complex revealed that the Hairless protein inserted deeply between two β sheets into the CTD of Su(H), thereby distorting the Su(H) structure in a way that precludes the binding of ICN to Su(H) [27]. Apart from the novelty of the binding, the analysis showed that Su(H) exclusively binds either ICN or Hairless. Earlier experiments have revealed that excess ICN was able to displace H from Su(H)-H complexes, indicating a certain degree of competition in the formation of activator or repressor complexes, respectively [26]. This competition most likely occurs in the cytoplasm, the place of ICN generation, because not only ICN, but also Hairless, shuttles Su(H) in the nucleus [3,28,29]. Accordingly, despite its function as a transcriptional repressor, Hairless is not strictly nuclear but also present in the cytoplasm, where it binds to Su(H) for nuclear import [28,29,30]. Nevertheless, as consequence of its nuclear export signal, it may also be involved in the shuttling of Su(H) protein between the nuclear and cytosolic compartments [29]. Su(H) protein not only relies on its co-factors ICN or Hairless for nuclear import. Moreover, its stability depends on complex formation, as in the absence of Hairless or Notch, Su(H) protein is considerably less abundant than in the wild-type [4]. Based on the crystal structure of the Su(H)-H repressor complex, three Leucine residues specific for H-binding were replaced by Alanine in Su(H)^LLL^, which consequently lead to a loss of Hairless binding [27]. Accordingly, Su(H)^LLL^ protein is barely detectable in the *Drosophila* tissue due to its inability to form a complex with H protein, indicating instability of the mutant protein [4].

Notch signaling activity is highly dose-sensitive: mutations in several Notch signaling components are haplo-insufficient, exhibiting a dominant phenotype, thereby facilitating genetic interaction studies [31,32,33]. For example, the Notch mutant wing phenotype is almost normalized by a second site mutation in the Hairless locus, implying that the ratio of activator to repressor should be ideally one to one [31,33,34]. Therefore, the question arose what happened if Hairless was unable to enter the nucleus. To achieve this scenario, a transmembrane domain was fused to Hairless in order to anchor Hairless protein at membranes, ideally confining it in the cytosol. Based on the hypothesis that Hairless binds Su(H) in the cytosol for nuclear import, in this experimental setting, Su(H) should be likewise trapped, thereby preventing the formation of repressor as well as activator complexes. The phenotypic consequences observed in the experiments support this working hypothesis.

## 2. Materials and Methods

### 2.1. Cloning of the Hairless Transmembrane Coding Sequence

The signal peptide (SP) and the transmembrane domain (TM) were derived from Delta (complete Delta cDNA in pIZ V5-His expression vector, provided by Delidakis [35]); the cloning strategy is depicted in Figure S1. The fragment coding for the signal peptide (M1 to T125) was excised by an *Eco* RI/*Kpn* I digest and subcloned into likewise digested pUASTattB vector [36] generating pUAST SP. The sequence coding for the transmembrane domain was PCR-amplified with upper primer 5′ GCG AGA GCC GAT *GGT ACC* ACC AAT 3′ with *Kpn* I site and lower primer 5′ CCC ACT GCC ATT GTG A*AA GCT T*GT G 3′ with *Hind* III site (codons T591 to T646). The *Kpn* I/*Hind* III digested PCR product was cloned into likewise digested pBT (Stratagene, La Jolla, CA, USA) generating pBT TM. Into the Hairless cDNA clone [33], an additional *Sal* I site was introduced at position of the stop codon via site directed mutagenesis using primer pair Up 5′ GAA TCT GTC AAA GCA C*GT CGA C*AT ACA CAC GCC 3′ and Low 5′ GGC GTG TGT AT*G TCG AC*G TGC TTT GAC AGA TTC 3′. In this *Sal* I site and the adjacent polylinker *Hind* III site, a Myc-tag plus stop codon derived from pESC-LEU yeast vector (Stratagene, La Jolla, CA, USA), was cloned to generate pBT Hairless-myc. Subsequently, a *Hind* III digest released a Hairless fragment including codon position S212 to the C-terminal Myc-tag and stop codon, subcloned into pBT TM to generate pBT TM H. TM H was released with *Kpn* I and *Xba* I and inserted into the likewise digested pUAST SP construct to obtain the final Delta SPTM-Hairless Myc (SPTM-H^myc^) pUAST attB transformation vector. The Hairless^myc^ cDNA and Hairless cDNA [26] were likewise cloned in pUAST attB and transgenic lines established for control expression.

### 2.2. Cloning of the GFP Transmembrane Coding Sequence

The GFP coding DNA fragment was derived from pEGEF-N1 vector (Clontech, Palo Alto, CA, USA) and cloned with *Sac* I/*Not* I into pBT vector (Stratagene, La Jolla, CA, USA). To secure the open reading frame with transmembrane sequence, GFP was excised with *Bam* HI/*Xba* I and recloned into *Bam* HI/*Xba* I digested pBT. This step was necessary to omit polylinker sequences of pEGEF-N1 vector and to inverse the orientation. This allowed the subsequent release of a *Hind* III/*Xba* I fragment for cloning in frame with pBT TM. Subsequently, a *Kpn* I/*Xba* I fragment was inserted into likewise opened pUAST SP to obtain the Delta SPTM-GFP pUAST attB transformation vector.

### 2.3. In Vivo Analysis

The pUAST attB constructs were all integrated at chromosomal position 68E using the PhiC31 integration system as described before [26,36]. Several strains were obtained for each construct that all behaved similarly. Tissue specific expression was induced with the Gal4/UAS system [37]. For driver lines, *omb*-Gal4 recombined with *vg*^BE^-lacZ [38], Bx-Gal4 (also named MS1096-Gal4), *pnr*-Gal4, and *sd*-Gal4 were used (http://flybase.org). pUAST attB SPTM-H^myc^ at position 68E was recombined with pUAST attB Su(H) and Su(H)^LLL^ constructs, respectively, located at chromosomal position 96E [4,27] to allow for co-overexpression. LacZ expression was detected by antibody staining using anti β-galactosidase (1:250) (clone 40-1a; developed by J.R. Sanes; obtained by Developmental Studies Hybridoma Bank, University of Iowa, IA, USA). Hairless was detected either with anti H antibodies from guinea pig (1:500) [39] or with anti-Myc-tag antibodies A4-1 from rabbit (1:1000) (Santa Cruz Biotechnology, Santa Cruz, CA, USA), to specifically detect transgene expression. Su(H) was detected with antibodies generated in rabbit (1:500) (Santa Cruz Biotech, Dallas, TX, USA) or rat [39]. For the detection of Lamin C (1:20) (LC28.26), Wingless (1:25) (4D4), Cut (1:25) (2B10), and β-galactosidase (1:250) (40-1a) mouse monoclonal antibodies were applied (developed by P.A. Fisher, S.M. Cohen, G.M. Rubin and J.R. Sanes, respectively; obtained by Development Studies Hybridoma Bank, University of Iowa, IA, USA). GFP was detected with rabbit anti-GFP (1:250) (Santa Cruz Biotech, Dallas, TX, USA) and Putzig with guinea pig anti-Pzg (1:1000) [40]. To outline cell membranes, filamentous actin was labeled with Rhodamine-coupled phalloidin (1:200; Molecular Probes, Eugene, OR, USA). The Golgi compartment was marked with mouse antibodies against the human p230 tran*s*-Golgi (1:500) (clone 15; BD Biosciences, San Jose, CA, USA) and goat antibodies against *Drosophila* Gmap *cis*-Golgi (1:1000) [41]. Secondary antibodies coupled with FITC, Cy3, and Cy5 (all 1:200) were purchased from Jackson Immuno-Research Laboratories (Dianova, Hamburg, Germany). Fluorescently labelled tissue was prepared as described earlier and mounted in Vectashield (Vector labs, Eching, Germany). Pictures were taken with a confocal microscope (BioRad, MRC 1024, Munich, Germany) linked to a Zeiss Axioskop using LaserSharp 2000TM software (Zeiss, Jena, Germany). Adult wings were dehydrated in ethanol, mounted in Euparal (Roth, Karlsruhe, Germany) and documented with an ES 120 camera (Optronics) linked to a Zeiss Axiophot (Zeiss, Jena, Germany), using Pixera Viewfinder Pro2.5 software (Pixera, Santa Clara, CA, USA). Adult flies were etherized and pictured with a table top scanning electron microscope JCM-5000 SEM (Nikon, Tokyo, Japan). Pictures were assembled using Image J (open source), Photo Paint, and Corel Draw software (Corel, Ottawa, Canada).

### 2.4. Western Blotting

pUAST attB SPTM-H^myc^ and H^myc^ flies were crossed to *da*-Gal4 for ubiquitous expression. Embryos were collected overnight at room temperature on apple juice plates, and dechorionated in 50% sodium hypochlorite bleach. Two hundred embryos each were homogenized on ice in 50 µL RIPA I buffer (300 mM NaCl, 100 mM Tris-HCl pH 7.5, plus one tablet Roche complete ULTRA protease inhibitor). After centrifugation, loading buffer was added to the supernatant, which was boiled before separation by SDS-PAGE, followed by Western blotting. Hairless was detected either with anti-H antibodies from guinea pig (1:500) [39] or with anti-Myc tag antibodies A4-1 from rabbit (1:1000) (Santa Cruz Biotechnology, Santa Cruz, CA, USA). Anti-rabbit or anti-mouse secondary antibodies from guinea pig coupled with alkaline phosphatase were used for detection (Jackson Immuno-Research, obtained from Dianova, Hamburg, Germany).

### 2.5. Statistical Evaluation

Area of female wings was determined using the freehand tool of Image J (open source). Bristles were counted in a determined area on female thoraces. Statistical analysis was conducted by Student’s *t* test: *** *p*  <  0.001 highly significant; ** *p*  <  0.01 very significant; * *p*  <  0.05 significant; not significant (ns) *p*  >  0.05. Graphs were compiled with Origin^®^2018b software from Origin lab (Northampton, MA, USA).

## 3. Results

### 3.1. Cloning Strategy

The aim of the cloning strategy was to obtain a cDNA coding for Hairless coupled N-terminally to a transmembrane domain as well a signal peptide necessary for integration into plasma membranes. Moreover, the Hairless protein should be in the cytosolic compartment.

**Figure 1:**
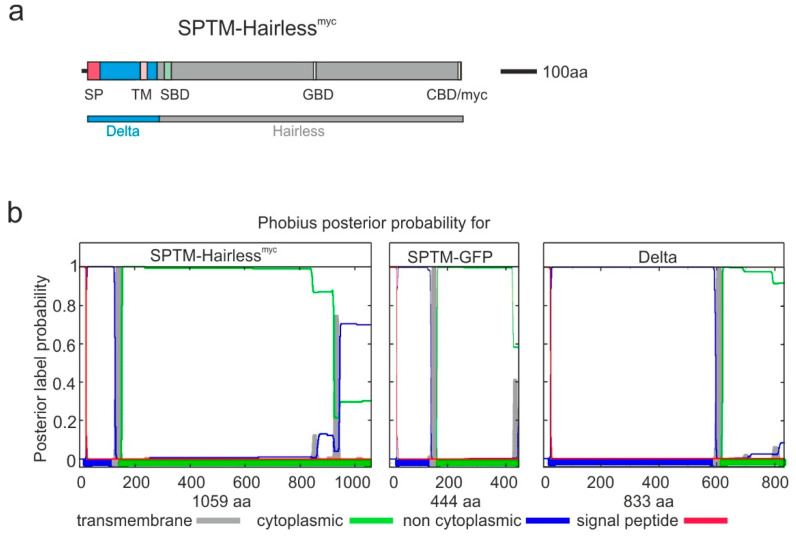
Construction of a membrane-anchored Hairless isoform. (**a**) Scheme of the SPTM-H^myc^ transgene. Delta protein (blue) containing the signal peptide (SP, magenta) and the transmembrane domain (TM, pink) was fused to Hairless protein (grey) containing the Su(H) binding domain (SBD, green), the Groucho binding domain (GBD), the binding domain for the C-terminal binding protein (CBD), as well as a C-terminal Myc-tag (myc). Scale corresponds to 100 amino acids (aa). This SPTM-H^myc^ construct was cloned into pUAST attB vector to be integrated at position 68E in the fly genome; (**b**) Plots showing predicted transmembrane domain and signal peptides established with the phobius database (http://phobius.sbc.su.se). The posterior label probability of signal peptide (red) and transmembrane (grey) is shown on the *y*-axis (1 = 100% probability). The *x*-axis represents the amino acids of the evaluated protein as well as the probability of the subcellular localization (green = cytoplasmic orientation.

Hairless possesses an internal ribosomal entry site (IRES) within the coding region, which leads to two main Hairless protein isoforms starting with the second and third Methionine, M2 and M3, respectively (Figure S1) [42,43]. Both isoforms contain all known functional Hairless domains: Suppressor of Hairless binding domain (SBD), Groucho binding domain (GBD), and C-terminal protein binding domain (CBD) [23,24,25], as well as the known nuclear import and export signals [29]. To avoid translation of a Hairless protein isoform without transmembrane domain by internal ribosomal entry, only the Hairless portion downstream of the IRES was fused with the transmembrane domain (Figure 1a and Figure S1).

For the transmembrane domain and the signal peptide, I choose that of Delta and not of Notch to avoid intra-membrane cleavage [35]. Cloning details are outlined in Figure S1: apart from the Delta transmembrane domain, which was obtained by PCR amplification including relevant restriction sites, it was possible to clone the construct using internal restriction sites. The resultant construct has an open reading frame starting 5′ with the signal peptide followed by sequences for the transmembrane domain and ending 3′ with a Myc-tag added to the Hairless coding sequences (Figure 1a and Figure S1a). To generate an appropriate control, GFP was fused in a similar way with the Delta transmembrane domain and signal peptide. The final constructs were sequence-verified. Both peptides are predicted transmembrane proteins with the major body in the cytosol by the combined transmembrane topology and signal peptide predictor Phobius [44] (Figure 1b). Using the pUAST attB vector, they were each inserted at position 68E with the PhiC31 integration system [36], generating UAS-SPTM-H^myc^ and UAS-SPTM-GFP, respectively. Protein sequences are shown in Figure S2. In vivo expression of SPTM-H^myc^ protein was verified in Western blots. To this end, ubiquitous protein expression was induced in embryos by crossing UAS-SPTM-H^myc^ with *da*-Gal4 (Figure S1b,c).

### 3.2. In Vivo Consequences of the Overexpression of SPTM-H^myc^ Protein

#### 3.2.1. Subcellular Localization of Overexpressed SPTM-H^myc^ Protein in Salivary Glands

**Figure 2:**
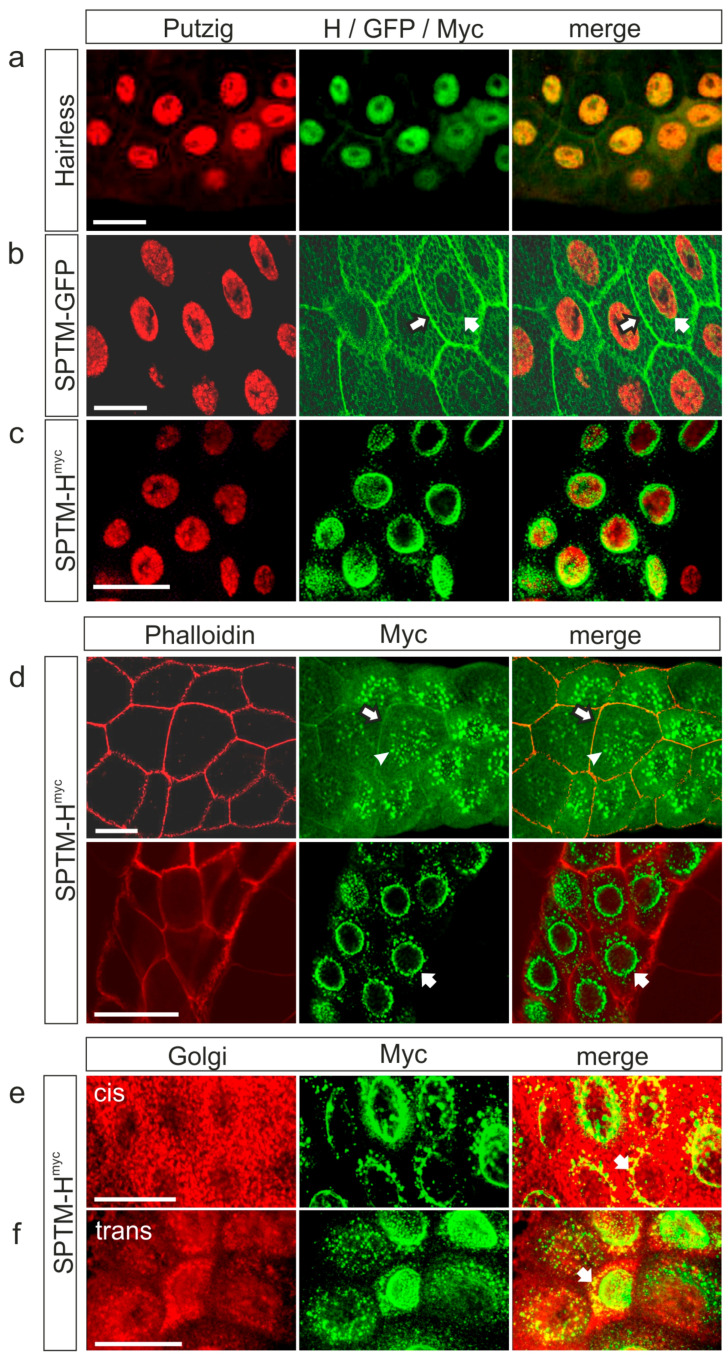
Subcellular localization of the given fusion proteins. Salivary glands from third instar larvae; expression of the given constructs was induced with *sd*-Gal4 and detected with respective antibodies against H (**a**), GFP (**b**), and Myc (**c**), labelled in green. Nuclei were marked with anti-Putzig (**a–c**); cell outlines with Rhodamin-coupled Phalloidin (**d**), *cis*-Golgi with anti-Gmap (**e**), and *trans*-Golgi with anti-p230 (**f**), labelled in red. Size bars represent 50 µm. (**a**) Wild-type H strongly accumulated in the nucleus; (**b**) SPTM-GFP protein was associated with the cell membrane (black-framed arrow) and the nuclear envelope (arrow); (**c**) No co-localization of SPTM-H^myc^ and Putzig is seen; instead, SPTM-H^myc^ is highly enriched at the nuclear envelope; (**d**) Upper panel: SPTM-H^myc^ protein is visible in a more punctuated pattern in the proximal part of the gland (example marked with arrowhead) and is detected also at the outer cell membrane (black-framed white arrow). Lower panel: a prominent staining at the nuclear envelope encircled by a corona of apparent vesicles (arrow) is present in a more proximal part frequently seen in same gland; (**e**,**f**) Co-localization of SPTM-H^myc^ protein (green) with the *cis*-Golgi marker Gmap (red) (**e**) and the *trans*-Golgi marker p230 (red) (**f**) is seen (overlap appears yellow in the merge, arrow).

The cells are very small in *Drosophila* larval tissue, which makes it difficult to detect the subcellular localization of proteins, especially if expected in the cell membrane. Therefore, expression of SPTM-H^myc^ was first induced in salivary glands of third instar larvae that contain giant cells and nuclei, using the driver line *sd*-Gal4. Since endogenous Hairless protein is primarily detected in the nucleus (Figure 2a) [28,29,30], the nuclear protein Putzig (Pzg) was used as a marker [40]. As expected, the overexpressed SPTM-GFP control protein was not nuclear but mainly associated with the cell membrane, however, was also seen in a meshwork throughout the cytosol (Figure 2b). In contrast to the wild-type Hairless protein, SPTM-H^myc^ was absent from salivary gland nuclei (Figure 2c). The most prominent staining was observed at the nuclear envelope (Figure 2c–e).

To confirm the expected membrane staining, cells were outlined by visualizing filamentous actin with phalloidin (Figure 2d). Again, the most prominent staining for SPTM-H^myc^ was at the nuclear envelope; however, it was also seen at the outer cell membrane. Staining of the plasma membrane seemed obscured by glue protein vesicles, and appears in a different focal plane than the nuclear envelope (Figure 2d) [45,46]. A co-staining with two different Golgi markers conforms to SPTM-H^myc^ present in the Golgi compartment as well as close to the endoplasmic reticulum (ER) (Figure 2e,f).

**Figure 3:**
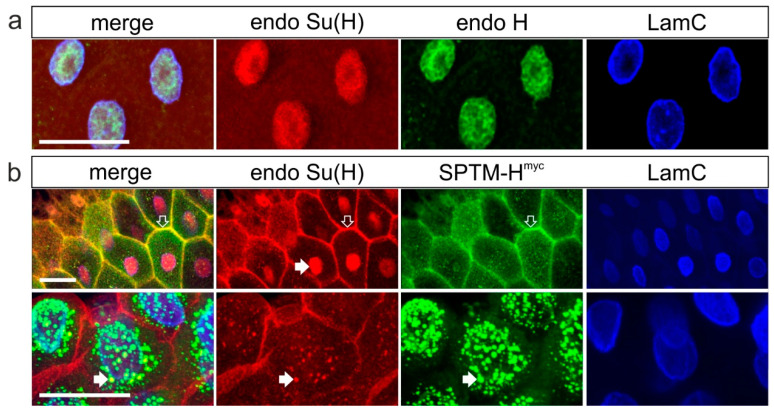
Su(H) protein co-localizes with SPTM-H^myc^ in salivary glands. (**a**) Endogenous expression of Hairless (H, green) and Su(H) (red) in salivary glands. Both proteins accumulate in the nucleus. The nuclear envelope is marked with Lamin C (LamC, blue); (**b**) co-localization of endogenous Su(H) (red) with ectopically expressed SPTM-H^myc^ protein (green). Upper panel: mostly localized in the nucleus (arrow), endogenous Su(H) is also present along the outer plasma membrane just like SPTM-H^myc^ (open arrows). Lower panel: a different focal plane reveals co-localization of endogenous Su(H) and SPTM-H^myc^ also along the nuclear periphery within the presumptive Golgi compartment (arrow) in a more distal portion of the salivary gland. Size bars represent 50 µm.

Next, a double staining of SPTM-H^myc^ with endogenous Su(H) was performed. Endogenous Su(H) is expected to accumulate in the nucleus (Figure 3a) [47]. If Su(H) is trapped by SPTM-H^myc^, a co-localization of both proteins is expected: in fact, co-staining was detected in the Golgi compartment, and along the plasma membrane to a lower extent (Figure 3b). However, a considerable portion of endogenous Su(H) protein was present in the nucleus, indicating incomplete anchoring by SPTM-H^myc^.

#### 3.2.2. Phenotypic Consequences Resulting from SPTM-H^myc^ Overexpression

**Figure 4:**
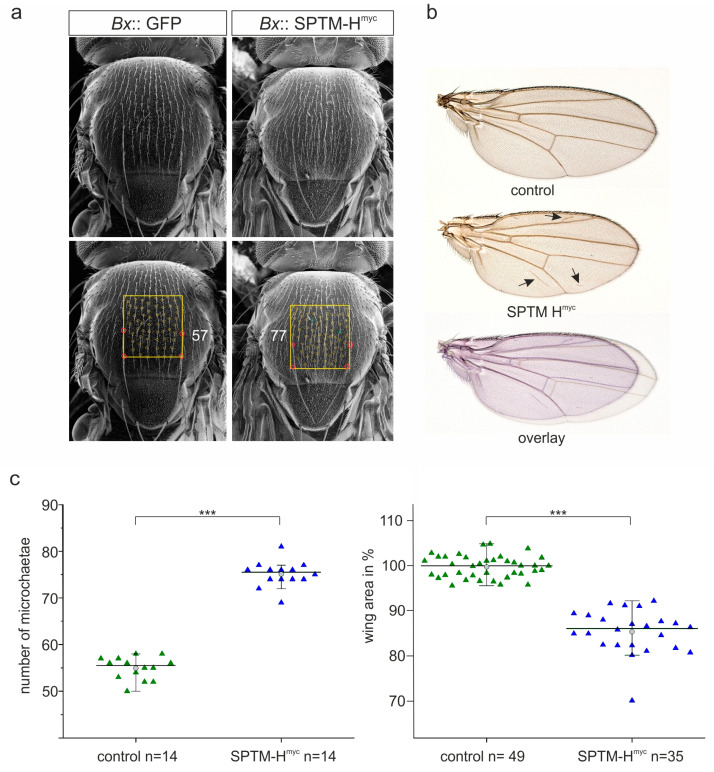
Consequences of SPTM-H^myc^ on adult development. Phenotypic analysis of adult flies upon SPTM-H^myc^ overexpression with *Bx*-Gal4 at 29 °C. *Bx*-Gal4 drives expression in the anlagen of wing and thorax [48]. (**a**) Scanning electron micrographs show thoraces of flies; GFP for control and SPTM-H^myc^, respectively, were overexpressed during development. Below, the same images illustrating data analysis: the area of bristle counts is outlined and the counted microchaetae are encircled in yellow (GFP 57, SPTM-H^myc^ 77 microchaetae). Red circles mark the anterior and posterior dorsocentral macrochaetae; (**b**) Wings of female flies overexpressing GFP (control) and SPTM-H^myc^, respectively. Note additional wing veins (arrow) and smaller wing size in the mutant. The latter is clearly seen in the overlay (GFP is shown in grey, SPTM-H^myc^ is colored in purple); (**c**) statistical graphs depicting total microchaetae counts (left panel) and wing area in % of control (right panel). Circle, mean value; horizontal bar, median; error bars, standard deviation. Sample size is given below (n). The differences between GFP control and SPTM-H^myc^ are highly significant (*p* < 0.001 ***) by Student’s *t*-test.

Next, the consequences on fly development were addressed: using the *Bx*-Gal4 driver line, UAS-SPTM-H^myc^ line was overexpressed in the anlagen of the meta-thorax and the wings [48]. Both tissues are known to be highly susceptible to a change in Notch signaling activity. In contrast to the SPTM-GFP control, SPTM-H^myc^ affected the development of mechano-sensory bristles on the thorax as well as the development of the wing at 29 °C (Figure 4). Notably, the number of microchaetae was elevated, which is a typical sign of reduced Notch activity (Figure 4a,c) [31,34]. For a quantification of bristle numbers, pictures of thoraces were taken by scanning electron microscopy, and the microchaetae were counted within a square between the intrascutal suture and the posterior dorsocentral macrochaetae in two independent experiments (Figure 4a). Whereas control flies displayed an average of about 55 microchaetae, flies overexpressing SPTM-H^myc^ featured 75 microchaetae on average (Figure 4a,c). This is a highly significant increase in bristle number of almost 37%. In addition, the wings of flies overexpressing SPTM-H^myc^ appeared smaller compared to control, and frequently contained additional wing vein material (Figure 4b). A quantification of wing size revealed a highly significant reduction of about 13% due to SPTM-H^myc^ overexpression (Figure 4c). As Notch activity regulates growth, this phenotype conforms to reduced Notch activity [49,50,51]. In sum, excessive SPTM-H^myc^ protein interferes with Notch activity, presumably by sequestering Su(H) protein within the cytoplasm by its membrane-anchor (Figure 3b), thereby limiting its availability for ICN, and hence for activator-complex assembly.

#### 3.2.3. Influence of SPTM-H^myc^ on the Activity of Notch Target Genes in Wing Imaginal Discs

**Figure 5:**
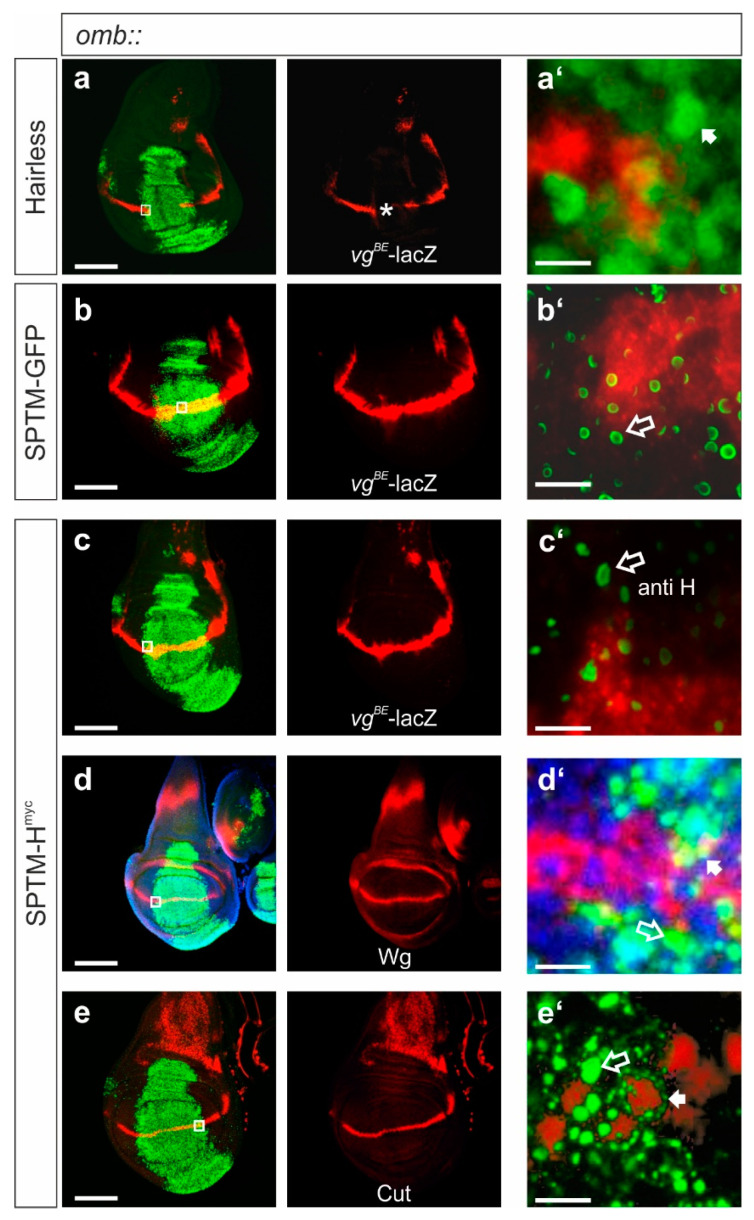
Effects of SPTM-H^myc^ overexpression on Notch target genes. Ectopic expression of the respective constructs was induced with *omb*-Gal4 in wing imaginal discs. (**a**) Expression of wild-type Hairless protein; (**b**) of SPTM-GFP, (**c–e**) and of SPTM-H^myc^ is shown in green. Expression of Notch targets, (**a–c**) the *vg^BE^*-lacZ reporter, (**d**) Wingless (Wg), and (**e**) Cut is shown in red. (**d**) Nuclei are marked in blue by anti-Pzg staining. (**a’–e’**) Large magnifications and selected stacks are shown. The area of the enlargements is depicted as the white square in (**a–e**). Size bars represent 100 µm in (**a–e**), and 5 µm in (**a’–e’**). (**a**) Whereas ectopic H repressed the *vg^BE^*-lacZ reporter along the dorso-ventral border (asterisk), SPTM-H^myc^ did not; compare (**c**) with control in (**b**). Moreover, little influence was noticed on Notch targets Wg or Cut (**d,e**). (**a’**) Note nuclear staining of H (arrow). (**b’**) Membrane-anchored SPTM-GFP, (**c’–e’**) and SPTM-H^myc^ proteins instead appear in a punctuated pattern, sometimes in circle-like vesicles (**b’**,**c’**; open arrow). These larger vesicles are separate from the nuclear envelope, which is obvious in the double staining of SPTM-H^myc^ with the nuclear protein Cut (red in **e’**). Compare also with enlargement in (**d’**, open arrow); Pzg staining served as nuclear marker (blue). The small arrow points to smaller dots in the nuclear periphery presumably corresponding to the ER (**d’**,**e’**). This is quite apparent in (**e’**), where the small dots of SPTM-H^myc^ fully encircle the nucleus (small arrow).

If the above assumption were correct, an interference of excessive SPTM-H^myc^ protein with Notch target gene activity would be expected. To address this idea, SPTM-H^myc^ was overexpressed just in the center of wing imaginal discs using the *omb*-Gal4 driver line, to allow for a comparison with adjacent wild-type cells. UAS-SPTM-GFP served as control. The following Notch targets were investigated: *vestigial* (using the *vestigial* boundary enhancer coupled to a Lac-Z reporter gene, *vg^BE^*-lacZ) [38] (Figure 5a–c), Wingless (Figure 5d), and Cut (Figure 5e). Overexpression of SPTM-H^myc^ did not alter the expression of the Notch target genes in the *omb* domain, in contrast to the effects of an overexpression of wild-type H (compare Figure 5a with Figure 5c–e), but rather resembled the expression of the GFP control (compare Figure 5b with Figure 5c). Whereas excessive H resulted in the expected loss of *vg^BE^*–lacZ reporter gene expression along the dorso-ventral boundary (Figure 5a) [26,52], SPTM-H^myc^ did not effect a similar loss, and the discs matched control (Figure 5b,c). Likewise, no effect on the expression of Wingless (Figure 5d) nor of Cut (Figure 5e) was observed. These results demonstrate that SPTM-H^myc^ cannot silence Notch target gene activity to a similar degree as the wild-type H protein in wing imaginal discs (Figure 5). However, SPTM-H^myc^ may ameliorate Notch activity as reflected by the defects seen in the adult flies (Figure 4). Presumably, ICN’s affinity to Su(H) is high enough to recruit adequate amounts for activator complex assembly to induce target gene expression. This may be even in exchange for binding to Hairless, since ICN is able to outcompete Hairless in vitro [26].

A detailed analysis of the samples at high magnification, however, confirmed the results from the salivary glands with a primary expression of SPTM-H^myc^ protein along the nuclear envelope (see enlargements in Figure 5a’–e’). SPTM-H^myc^ protein accumulated in a punctuated pattern (or vesicles) within the *omb* expression domain (Figure 5c’–e’), a pattern clearly different from the ectopic expression of a wild-type Hairless protein, which is enriched in the nucleus (Figure 5a’). In single confocal sections, SPTM-H^myc^ protein was detected in circles, similar to SPTM-GFP (Figure 5b’,c’). In stacked images, SPTM-H protein appeared as dots (Figure 5d’,e’), encircling the nucleus compared to the nuclear protein Cut, presumably corresponding to the ER (Figure 5e’, small arrow), whereas the larger dots may correspond to the Golgi compartment (Figure 5d’,e’, open arrow). A co-staining with the nuclear marker Pzg showed little or no overlap of the dots with the nuclei; an apparent overlay is a result of the summary of the stacks (Figure 5d’). Using cytoplasmic Wingless expression as an additional marker, a clearly separate staining from the Pzg nuclear expression was noted (Figure 5d’, arrow), however, a frequent overlap with SPTM-H^myc^ was seen as yellow mix color (Figure 5d’, open arrow).

### 3.3. Effects of a Combined Overexpression of SPTM-H^myc^ and Su(H)

In the next series of experiments, UAS-SPTM-H^myc^ was overexpressed in combination with Su(H). Several questions were addressed: firstly, is Su(H) co-localized with SPTM-H^myc^ as predicted? Secondly, what are the effects on Notch target gene expression? Thirdly, what are the phenotypic consequences on fly development? Finally, the consequences of a combination with the Su(H)^LLL^ mutation deficient for Hairless-binding was investigated in comparison to the wild-type Su(H) [4,27]. Here, the expectation was that SPTM-H^myc^ should not affect Su(H)^LLL^ activity due to a lack of binding.

#### 3.3.1. Co-localization of SPTM-H^myc^ and Su(H) in Salivary Glands

**Figure 6:**
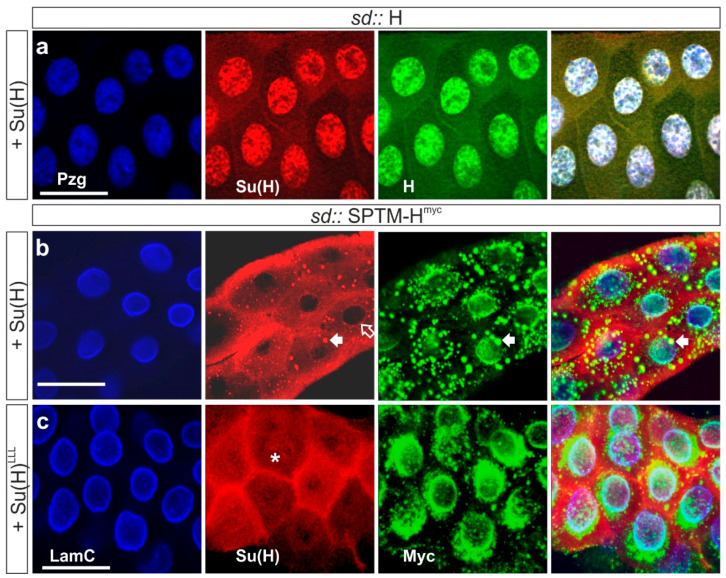
Combined overexpression of SPTM-H^myc^ with Su(H) in salivary glands. Ectopic expression of the given UAS constructs was induced in salivary glands using *sd*-Gal4. The nucleus is marked in blue with Putzig (Pzg) (**a**), and the nuclear envelope with Lamin C (LamC) in (**b**,**c**). Hairless is shown in red and Su(H) in green. The size bars represent 50 µm. (**a**) Note very strong nuclear accumulation of both wild-type Hairless and Su(H) proteins; (**b**) Su(H) co-localized with SPTM-H^myc^ in the presumptive Golgi (arrows). It appeared nearly absent from nuclei (open arrow); (**c**) H-binding defective Su(H)^LLL^ mutant protein appears evenly distributed and is also detected within the nucleus (asterisk). No co-localization with SPTM-H^myc^ was observed.

As shown earlier, Su(H) relies on Hairless for nuclear entry, closely following H protein distribution in wild-type and experimental settings [28,29]. Therefore, in co-overexpression experiments of wild-type Hairless with Su(H), both proteins are almost completely nuclear (Figure 6a). Co-localization studies were performed in salivary glands of third instar larvae to take advantage of the huge size of the polytene cells. To this end, the transgenes UAS-SPTM-H^myc^ and UAS-Su(H) were recombined to be jointly overexpressed in salivary glands using *sd*-Gal4. A large, albeit not complete overlap of Su(H) and SPTM-H^myc^ proteins was noted (Figure 6b). Notably, there was a clear overlap in the punctuated staining along the nuclear envelope, presumably corresponding to the ER and Golgi compartment (Figure 6b, arrow). Curiously, Su(H) was virtually absent from the nucleus (Figure 6b, open arrow). In addition, a weak cytoplasmic staining was detected overall, presumably corresponding to free Su(H) not anchored by SPTM-H^myc^. In this experiment, SPTM-H^myc^ was detected with anti-Myc antibodies to display expression of the SPTM-H^myc^ transgene only, and not be confused by wild-type H protein.

Co-overexpression of SPTM-H^myc^ with Su(H)^LLL^ was according the expectations: explained by the lack of binding of the two proteins, no co-localization was observed. Instead, Su(H)^LLL^ protein was evenly distributed in the cytoplasm of salivary glands cells, whereas the punctuate-pattern typifying SPTM-H^myc^ protein was present in the nuclear periphery (Figure 6c). In contrast to Su(H), the mutant Su(H)^LLL^ protein was also weakly detected in the nucleus (asterisk in Figure 6c), which is attributed to Notch-mediated nuclear import [2,29].

#### 3.3.2. Consequences of the Co-Expression of SPTM-H^myc^ and Su(H) on Notch Target Gene Expression

**Figure 7:**
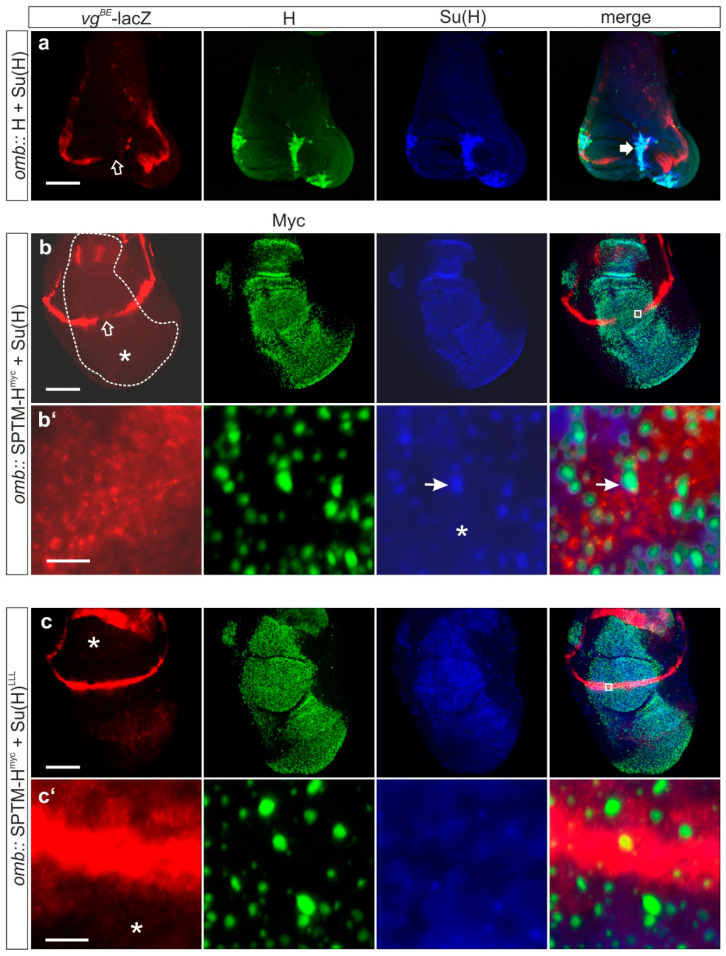
Influence of the combined overexpression of SPTM-H^myc^ and Su(H) on Notch reporter gene expression. Ectopic expression of the given UAS constructs was induced in imaginal discs using *omb*-Gal4. Expression of the *vg^BE^*-lacZ reporter is shown in red; of Hairless in green, and of Su(H) in blue. (**a**) In combination, wild-type Hairless and Su(H) induce extreme repression of Notch activity, reflected by the near complete loss of tissue (thick arrow) and a strong repression of the *vg^BE^*-lacZ reporter (open arrow); (**b**) The co-overexpression of SPTM-H^myc^ with Su(H) caused a gap in the expression of the *vg^BE^*-lacZ reporter along the dorso-ventral boundary (open arrow). In addition, the reporter was weakly induced throughout the *omb*-expression domain (asterisk). Note overgrowth of the affected tissue. The Su(H) staining largely followed the SPTM-H^myc^ staining (**b’**, arrow points to one example), however, appears less distinct and more uniform (**b’**, asterisk); (**c**) Overexpression of the H-binding defective Su(H)^LLL^ mutant protein induced an even more pronounced overgrowth, however, did not induce ectopic expression of the *vg^BE^*-lacZ reporter (asterisk), nor a repression along the boundary. Overall, Su(H)^LLL^ protein was present at a remarkably low level and showed no overlap with SPTM-H^myc^ (**c’**). Size bars represent 100 µm (**a–c**), and 5 µm (**b’**,**c’**), respectively. The area of enlargement is depicted as a white square in the merge picture of (**b**) and (**c**).

Notch target gene expression was analyzed in wing imaginal discs, overexpressing SPTM-H^myc^ plus Su(H) under the control of *omb*-Gal4, thereby allowing a comparison of wild typical and affected tissue. Expression of *vg^BE^*-lacZ [38] (Figure 7), of Wingless (Supplementary Figure S3), and Cut (Supplementary Figure S4) were investigated. Two opposing effects were noted. Whereas target gene expression vanished in the center of the *omb* expression domain, the entire domain expanded, displaying a faint overall protein expression (Figure 7b,b’, Supplementary Figures S3–S5). Downregulation of Notch target gene expression is a clear result of a repression of Notch activity. Overgrowth of the expression domain, and induction of expression, however, is in contrast a sign of a gain of Notch activity. The latter may be a result of excessive Su(H) protein, since similar phenotypes were observed before with sole Su(H) overexpression [26,52]. Accordingly, no super-repression was achieved, which is typical of the combined overexpression of wild-type Hairless together with Su(H) [23,26,52,53]. In this case, a severe tissue loss is observed because of a strong repression of Notch activity (Figure 7a).

Moreover, whereas the Hairless-Su(H) repressor complex was mostly nuclear, SPTM-H^myc^ and Su(H) proteins were not but rather co-localized in the dotted structures described above (Figure 7b,b’, Figures S3a and S4a). Accordingly, no such co-localization was observed in the combined overexpression with Su(H)^LLL^ lacking the Hairless-binding site (Figure 7c,c’ and Figure S4b). Since both combined overexpression settings resulted in a similar overgrowth of the disc (Figure S5), I conclude that excess Su(H) increased the availability for activator complex formation sufficient for increased Notch activity. Notably, no altering of Notch targets *vg^BE^*–lacZ or *wg* was observed when Su(H)^LLL^ was overexpressed together with SPTM-H^myc^, in contrast to wild-type Su(H) (Figure 7c,c’). I propose that SPTM-H^myc^ is able to sequester wild-type Su(H) but not Su(H)^LLL^, thereby reducing availability of Su(H) protein, thereby limiting Notch activity.

**Figure 8:**
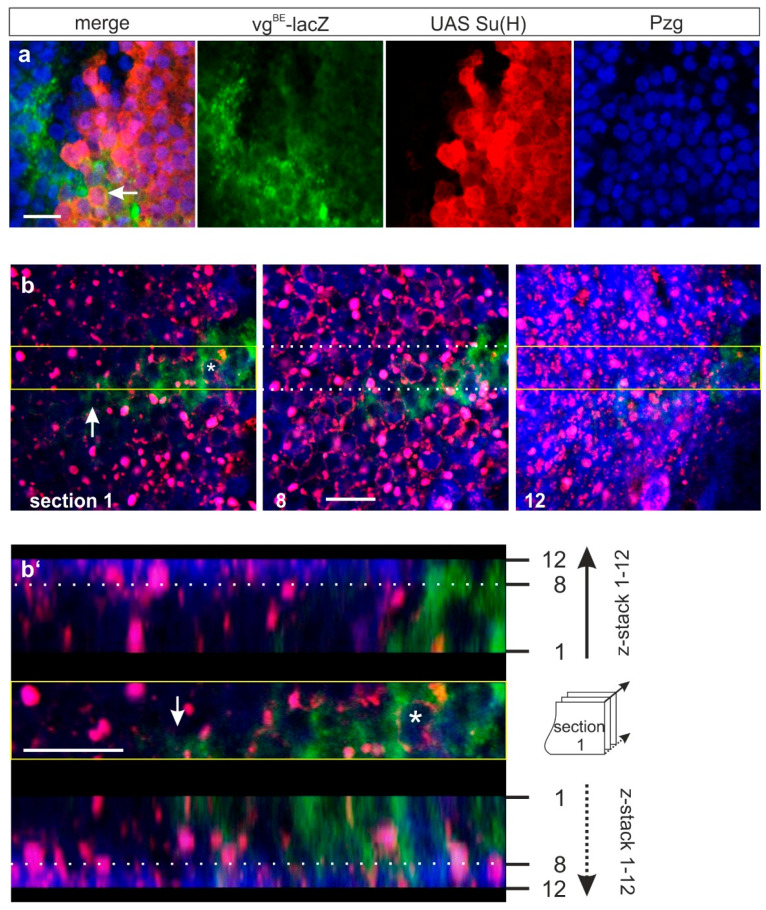
Co-localization of concurrently overexpressed SPTM-H^myc^ and Su(H) protein. Combined overexpression of SPTM-H^myc^ and Su(H) in wing discs using *omb*-Gal4. Enlargements show details of subcellular expression; the size bars represent 10 µm. (**a**) Su(H) protein (red) accumulated outside of the nucleus marked with Pzg antibodies (blue), overlapping *vg^BE^*-lacZ staining (green) (arrow in the merge picture); (**b**,**b’**) Z-stack analysis to reveal the uneven distribution of the Su(H) protein (blue). SPTM-H^myc^ was detected in red, and the *vg^BE^*-lacZ reporter in green. Three sections are shown, representing the upper, middle, and lower part (1, 8, 12) of a 4.8 µm thick tissue. The area marked by a yellow and dotted line was compiled to a *Z*-stack shown in (**b’**). Note near-complete overlap of SPTM-H^myc^ and Su(H) protein (appears pink in the merge) up to section 8. In sections 9 to 12, Su(H) is seen uniformly also outside of SPTM-H^myc^ dots, and appears as a blue smear. The asterisk labels a nucleus, encircled by SPTM-H^myc^ plus Su(H) protein. The cytoplasmic *vg*^BE^-lacZ staining (green) is repressed in the *omb*-domain (arrow in **b**,**b’**).

The subcellular distribution of SPTM-H^myc^ and Su(H) was analyzed in greater detail (Figure 8). Clearly, both proteins accumulated in the periphery of nuclei (Figure 8a, arrow and Figure 8b, asterisk). Whereas SPTM-H^myc^ appeared to be there exclusively, Su(H) was less well-distinguished and more evenly distributed also in the cytoplasm (Figure 8a). This distribution was especially well-dissolved in comparing different confocal sections in imaginal wing discs (Figure 8b,b’). The abundance of Su(H) protein differed from apical to basal level: it was primarily enriched in lower sections (8–12), overlapping and adjacent to SPTM-H^myc^ (Figure 8b’). In more apical sections, Su(H) was fully co-localized with SPTM-H^myc^, seen as pink mix of Su(H) blue and SPTM-H^myc^ red colors (Figure 8b’). The cytoplasmic *vg*^BE^-lacZ staining (green, asterisk) was repressed in the *omb* domain (Figure 8b,b’).

#### 3.3.3. Phenotypic Consequences of Co-Overexpression of SPTM-H^myc^ and Su(H)

**Figure 9:**
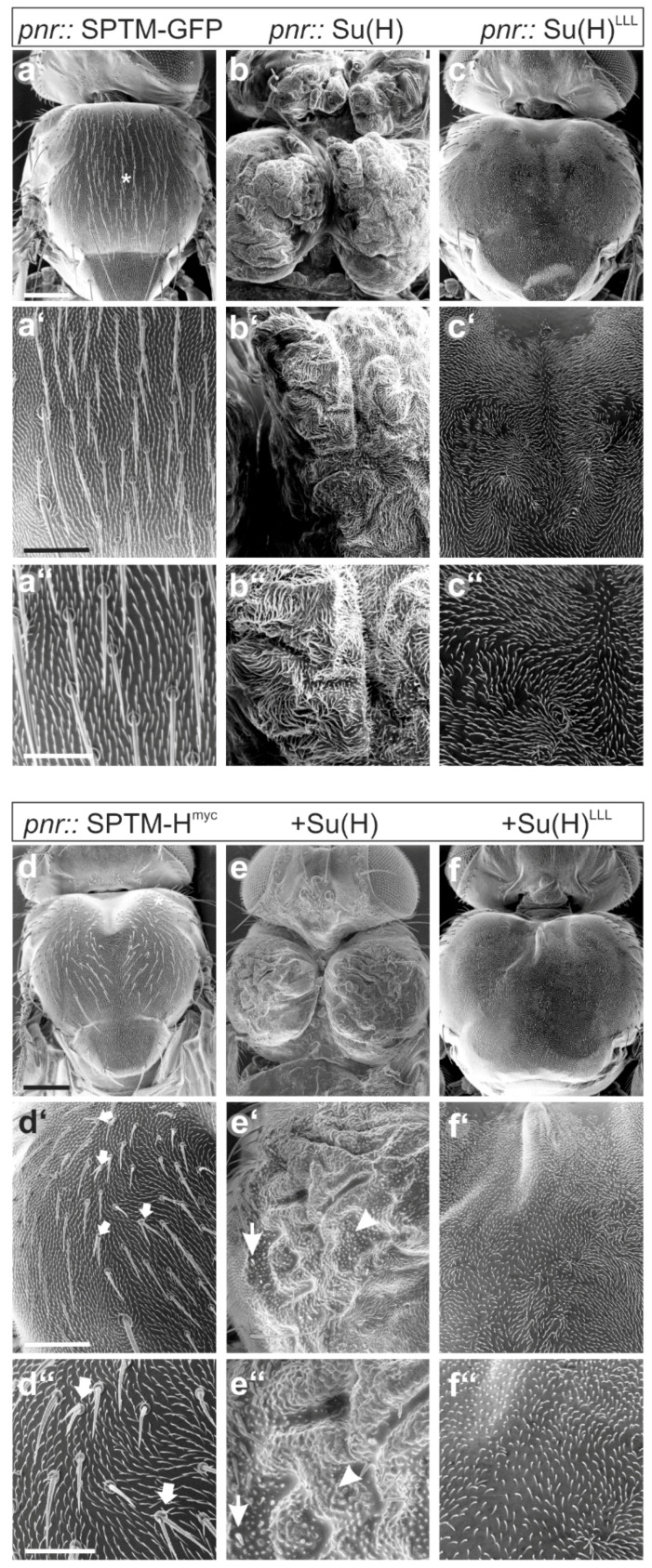
Developmental consequences of the combined overexpression of SPTM-H^myc^ and Su(H). Scanning electron micrographs of animals, where the given UAS constructs were induced during development using *pnr*-Gal4 at 25 °C. (**a–a’’**) SPTM-GFP control. Note slightly erratic bristle arrangement (asterisk), but normal structure; (**b–b’’**) wild-type Su(H) caused massive over-proliferation with heavily folded tissue; the whole body is split into two halves separated by a very deep cleft. No bristles developed. The animals died as pharate adults, and were dissected from the pupal case; (**c–c’’**) ectopic expression of Su(H)^LLL^ caused a massive overgrowth and a complete loss of chaetae without affecting tissue fusion; (**d–d’’**) the overexpression of SPTM-H^myc^ affected growth, resulting in a furrow in middle of the thorax and disturbing the orientation of the microchaetae. Moreover, bristles were duplicated, and a partial socket to shaft transformation was observed (small arrows in **d’**,**d’’**); (**e–e’’**) overexpressed in combination with Su(H), the flies hatched but displayed a deeply cleft thorax with strongly folded, over-proliferated tissue. Small bulges on the surface may be rudiments of bristle structures (arrowhead), as remains of shafts were present at the boundary to unaffected tissue (**e’**,**e’**’, arrow); (**f–f’’**) the co-overexpression of SPTM-H^myc^ with Su(H)^LLL^ was nearly indistinguishable from the sole Su(H)^LLL^ overexpression: the thorax was overgrown with no bristle structure left. Scale bar, 200 µm in (**a–f**), 100 µm in (**a’–f’**), and 50 µm in (**a’’–f’’**).

The consequences of the combined overexpression of SPTM-H^myc^ and Su(H) on fly development were analyzed. The driver line *pnr*-Gal4 was used, driving expression in the central part of the developing notum and the head [54]. Several defects were noted, mostly affecting growth and bristle formation on the thorax. Overexpression of the SPTM-GFP control with *pnr*-Gal4 at 25 °C caused a mild disorganization of the bristles on the thorax (asterisk in Figure 9a) without affecting bristle structure (Figure 9a’,a’’). Su(H) overexpression impeded fusion of the imaginal discs, resulting in a deep cleft in the center of the thorax, presumably as a result of extreme over-proliferation of the tissue (Figure 9b–b’’). In addition, bristle formation was completely abolished. These flies were unable to hatch and had to be freed from the pupal case for analysis. The ectopic expression of the Su(H)^LLL^ mutant, which is unable to bind Hairless, also induced massive over-proliferation accompanied by a complete bristle loss, however, did not affect dorsal fusion nor eclosion (Figure 9c–c’’).

A dorsal cleft resulted from the overexpression of SPTM-H^myc^ (Figure 9d). Moreover, bristle defects were observed, for example, bristle duplications or a partial transformation of socket to shaft cell type affecting microchaetae, as well as loss of macrochaetae (Figure 9d–d’’). In combination with Su(H), SPTM-H^myc^ impeded dorsal fusion, resulting in a deep dorsal cleft on the thorax. The tissue appeared folded from over-proliferation, however, not well-differentiated, accompanied by a complete absence of all bristles (Figure 9e). At best, some remnants of bristles or trichomes were spotted (Figure 9e’,e’’). However, the flies hatched, whereas the sole overexpression of Su(H) caused pharate lethality. This is a remarkable rescue, corroborated by the less pronounced over-proliferation (compare Figure 9b,e), that demonstrates the ability of SPTM-H^myc^ to sequester Su(H), thereby mitigating its activity. In contrast, the co-expression of SPTM-H^myc^ with Su(H)^LLL^ was not much different from the sole Su(H)^LLL^ overexpression: a dramatic overgrowth of the thorax, which was completely naked, was observed (Figure 9f–f’’). This result confirmed my prediction, since SPTM-H^myc^ is unable to bind and trap Su(H)^LLL^ and, hence, should not influence its activity. Overall, the experiments show that overexpression of SPTM-H^myc^ confounds the activity of ectopic Su(H), which is apparent when comparing the effects with Su(H)^LLL^, that is unaffected by SPTM-H^myc^.

## 4. Discussion

In this study, the consequences of a Hairless protein lacking the capacity to enter the nucleus were examined. By fusing Hairless to a transmembrane domain, the protein was immobilized at membranes. The type I transmembrane domain and the N-terminal signal peptide were taken from the Delta protein [55], fusing Hairless C-terminally to be directed into the cytoplasm. A Myc-tag allowed the specific detection of the subcellular localization of SPTM-H^myc^ protein, clearly distinguishable from the endogenous Hairless protein. In contrast to the wild-type protein, SPTM-H^myc^ is not nuclear [28,29,30]. Instead, it primarily accumulates at the periphery of the nucleus along the nuclear envelope. The punctuate pattern suggests enrichment in the endoplasmic reticulum, whereas larger dots further apart may correspond to the Golgi compartment, confirmed by an overlap of SPTM-H^myc^ with two Golgi markers. Accumulation at the outer plasma membrane was less pronounced, though. Nevertheless, the expression pattern demonstrates that SPTM-H^myc^ is anchored at membranes as desired.

**Figure 10:**
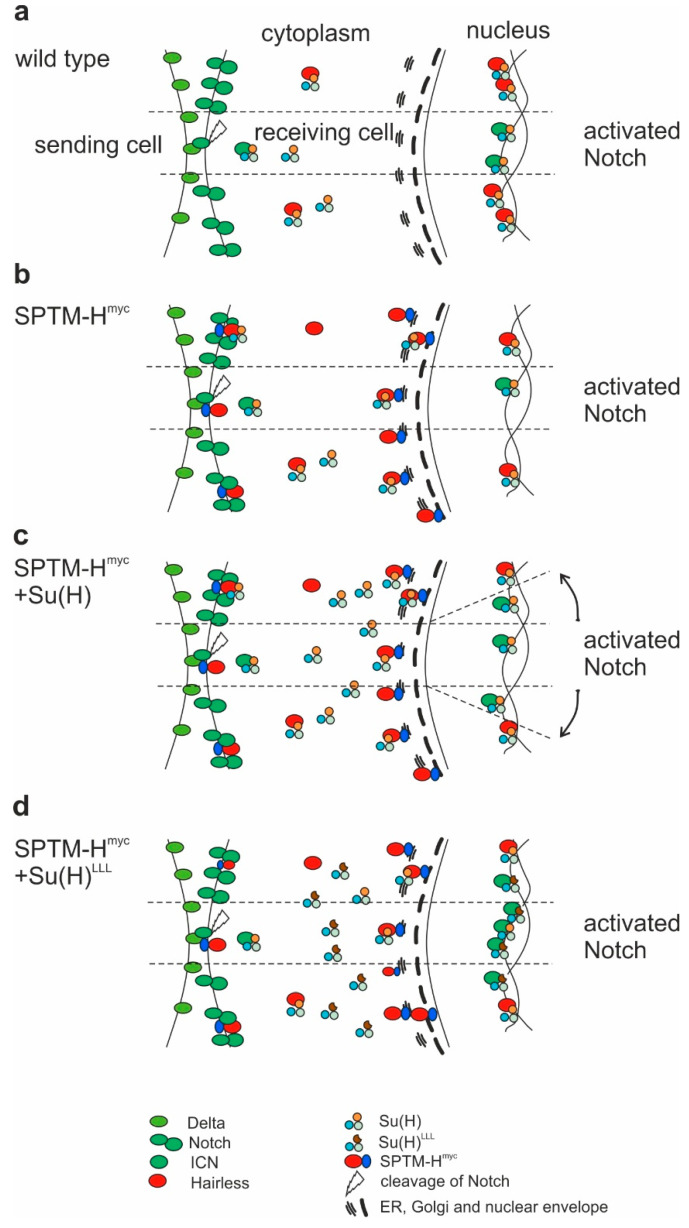
Model of SPTM-H^myc^ activity. (**a**) Wild-type situation of Notch signaling pathway: ligands like Delta activate Notch, releasing the intracellular Notch domain (ICN). On its way to the nucleus, ICN binds to Su(H) in the cytoplasm. Together with other activator molecules, this complex activates the Notch target genes. Outside the Notch activation domain (dashed lines), the process is antagonized by Hairless, which binds Su(H) in the cytoplasm and transports it in the nucleus, where, together with co-repressors, it forms a repressor complex to silence Notch target genes; (**b**) SPTM-H^myc^ integrates into membranes (cell membrane and ER and/or Golgi), sequestering freely available Su(H). Consequently, less activator and repressor complexes are present on the DNA. Since the ratio between activation and repression remains almost unchanged, little phenotypic consequences are observed. Yet, a slight downregulation of Notch activity indicates reduced availability of Su(H) for recruitment by ICN; (**c**) Overexpression of both, SPTM-H^myc^ and Su(H), gives a mixed result: repression of Notch targets at places of highest Notch activity, and activation of Notch targets elsewhere. The former may reveal insufficient availability of Su(H) due to sequestration by SPTM-H^myc^. The latter may result from recruitment of ectopic Su(H) into activator complexes by spurious levels of ICN in cells that are normally repressed by H activity; (**d**) Su(H)^LLL^ is deficient for the binding of H, here shown by a defective C-terminal domain (brown and clipped, instead of a full orange circle). Ectopic Su(H)^LLL^ hence acts independently of SPTM-H^myc^, causing a much higher Notch activation than in (**c**). Note that SPTM-H^myc^ is still able to sequester wild-type Su(H), yet affecting likewise activator and repressor complexes as in (**b**).

Indeed, overexpression of membrane-anchored SPTM-H^myc^ resulted in a downregulation of Notch activity. This effect was predicted in case of sequestering Su(H) by Hairless at the membrane, reducing its availability for activated Notch ICN, and hence activator complex formation and nuclear import (Figure 10a,b). The data confirm earlier findings that binding between Hairless and Su(H) indeed occurs in the cytosol before the nuclear translocation of the two proteins by Hairless [28,29]. Whether the entire repressor complex pre-assembles in the cytosol, or whether the co-repressors Gro and CtBP are recruited in the nuclear compartment, remains unknown. The same holds true for the activator complex: whereas it is clear that ICN binds Su(H) in the cytoplasmic compartment, assembly of the activator complex may occur only on the DNA [3,18,56,57,58].

As predicted by a downregulation of Notch activity, SPTM-H^myc^ overexpression affected tissue growth, as well as the formation of mechano-sensory organs and wing veins (Figure 4). It should be noted, however, that the intimate relationship between Notch and EGFR (epidermal growth factor receptor) signaling pathways opens up the possibility that some of the phenotypes or parts thereof result from an influence of SPTM-H^myc^ on EGFR signaling activity [59,60,61]. Notably, the ectopic veins induced by SPTM-H^myc^ overexpression (Figure 4b) could as well result from a gain of EGFR function [62,63,64,65,66]. Similarly, both tissue growth and apoptosis are regulated by EGFR as well [49,50,51,59,67,68,69].

The effects of the concurrent overexpression of SPTM-H^myc^ and Su(H), however, are less clear, and quite different from the ectopic expression of the wild-type proteins. The latter combination induces massive cell death in the affected tissues in addition to a strong repression of Notch target genes [26,52,53,69]. In contrast, the co-expression of Su(H) with SPTM-H^myc^ lead to a strong over-proliferation and expansion of Notch target gene expression on the one hand, and to a repression of Notch targets along the dorso-ventral boundary, on the other hand. Hence, a mixed response was observed: a more general activation of Notch signaling output, and a rather specific repression at places of highest Notch activity (Figure 7, Supplementary Figures S3 and S4). One possible explanation is the following: within the zone of highest Notch activation, i.e., along the dorso-ventral boundary, Su(H) levels are limiting for full Notch activity, because of the sequestering by membrane anchored Hairless (Figure 10b). Accordingly, a decrease of Notch target gene expression is expected. Outside of the Notch-activity zone, ectopic Su(H) becomes now available for activator-complex assembly, resulting in a weak overall activation of Notch target genes (Figure 10c). In the normal context, Su(H) is inhibited by endogenous Hairless within this area (Figure 10a), such that spurious levels of activated Notch ICN cannot induce the target genes. In the Su(H) overexpression context, however, endogenous Hairless levels do not suffice to limit Su(H) availability and apparently, neither does ectopic SPTM-H^myc^. The low level of activation hints at the fact that SPTM-H^myc^ sequesters Su(H) to a large degree, but not completely. Excessive Su(H), however, is able to join with spurious ICN to activate Notch targets at low level overall.

Why then does SPTM-H^myc^ not intercept Su(H) more strongly, as both are overexpressed concurrently? Perhaps not all of SPTM-H^myc^ integrates in the membrane, or the excess of membrane-coupled Hairless protein releases an unfolded protein response, albeit no signs of increased apoptosis were observed as expected in case of ER stress [70]. Alternatively, the ICN-Su(H) complex is more stable than the SPTM-H^myc^-Su(H) complex, allowing ICN to compete for Su(H) binding. This possibility is supported by the observation that Notch can displace Hairless from Su(H)-Hairless complexes in vitro, despite similar binding affinities in the nanomolar range [26].

In contrast, co-expression of SPTM-H^myc^ and Su(H)^LLL^ resulted in a different outcome. As Su(H)^LLL^ lacks Hairless-binding, increased activator complex formation without repression is expected as a result from excessive Su(H) availability (Figure 10d). Accordingly, there were little differences regarding the over-proliferation of wing discs with the *omb*-Gal4 driver or of thoraces with the *pnr*-Gal4 driver (Figure 7 and Figure 9, Supplementary Figures S4 and S5). No silencing of Notch target genes was observed, however, but rather an enhancement, although SPTM-H^myc^ may sequester wild-type Su(H) (Figure 10d).

Adult phenotypes support the interpretation that Su(H)^LLL^ largely bypasses interception of SPTM-H^myc^, in contrast to wild-type Su(H). Yet, they also revealed an interesting difference between the wild-type Su(H) and the H-binding defective Su(H)^LLL^ isoforms independent of their interaction with SPTM-H^myc^: whereas both isoforms induced marked over-proliferation within the thorax, Su(H) but not Su(H)^LLL^ impeded dorsal fusion (Figure 9). Dorsal fusion defects may result from interfering with Notch activity and/or from induction of apoptosis [71,72]. In this case, dorsal fusion defects resulting from sole Su(H) overexpression may be caused by repression of Notch rather than activation, hinting at its Janus-faced role as activator and equally as repressor of Notch targets [5,25,26,27,53,73]. Similar defects were also induced by the sole overexpression of Hairless [74] and likewise of SPTM-H^myc^ (Figure 9), reflecting the limitation of high levels of Notch activity due to reduced Su(H) availability. Accordingly, dorsal fusion was not improved by the combined overexpression of Su(H) with SPTM-H^myc^, whereas the over-proliferation of the affected tissue was diminished. Over-proliferation, however, is a clear sign of increased Notch activity [50]. These mixed phenotypes, resulting from concurrent overexpression of SPTM-H^myc^ and Su(H), are reminiscent of the Notch target gene response in imaginal discs—repression at positions of highest Notch activity and induction elsewhere. The defects in tissue differentiation could be a result of either gain or loss of Notch activity. Whether the phenotypic differences can be solely explained by the presented idea, namely that sequestration of Su(H) by SPTM-H^myc^ chops the peaks of highest Notch activity, but allows for a general increase of Notch activity outside the normal range, is an interesting question for the future.

## 5. Conclusions

In this work, the major Notch antagonist, Hairless, was expressed as a membrane-anchored protein isoform, impeding its normal function as a transcriptional co-repressor of Notch target genes. Histological analyses demonstrate that membrane-anchored SPTM-H^myc^ and Su(H) co-localize, indicating that Su(H) is sequestered and less available for the binding to activated Notch. Accordingly, lowered Notch signaling activity affected lateral inhibition and growth. In contrast, the combined overexpression of Su(H) and SPTM-H^myc^ enforced Notch signaling activity, reflected by the over-proliferation of tissue and a ubiquitous induction of Notch target gene expression. At the same time, Notch targets were repressed at their normal place of expression, raising the possibility that here, the threshold of highest Notch activity was no longer reached, presumably, because SPTM-H^myc^ trapped Su(H), reducing its availability. The work emphasizes earlier findings that Su(H)-Hairless protein complexes are formed in the cytoplasm, and that the binding to Hairless influences the availability of Su(H) for recruitment by activated Notch ICN.

## Supplementary Materials

The following are available online at https://www.mdpi.com/2073-4425/11/11/1315/s1, Figure S1: Construction of the *SPTM-H^myc^* transgene, Figure S2: Sequences of SPTM-H^myc^ and SPTM-GFP, Figure S3: Effect of the combined activity of SPTM-H^myc^ and Su(H) on Wingless expression, Figure S4: Effect of the combined activity of SPTM-H^myc^ and Su(H) on Cut expression, Figure S5: Effects on the size of the *omb*-expression domain. References [75] is cited in the supplementary materials.
