# Supplementary material for: Membrane-Anchored Hairless Protein Restrains Notch Signaling Activity
Source: Genes (Basel). 2020 Nov 6;11(11):1315. doi: 10.3390/genes11111315 (PMC7694644; doi:10.3390/genes11111315)
Supplement: Supplementary file 1 [file genes-11-01315-s001.pdf]

Supplemental Figures to

Membrane-anchored Hairless protein restrains Notch signaling activity

Maier D.
University of Hohenheim, Institute of Biology, Dept. of General Genetics, Garbenstr. 30, 70599 Stuttgart, Germany

Figure S1: Construction of the *SPTM-H^{myc}* transgene
Figure S2: Sequences of SPTM-H^{myc} and SPTM-GFP
Figure S3: Effect of the combined activity of SPTM-H^{myc} and Su(H) on Wingless expression
Figure S4: Effect of the combined activity of SPTM-H^{myc} and Su(H) on Cut expression
Figure S5: Effects on the size of the *omb*-expression domain

Figure S1: Construction of the *SPTM-H^myc^* transgene

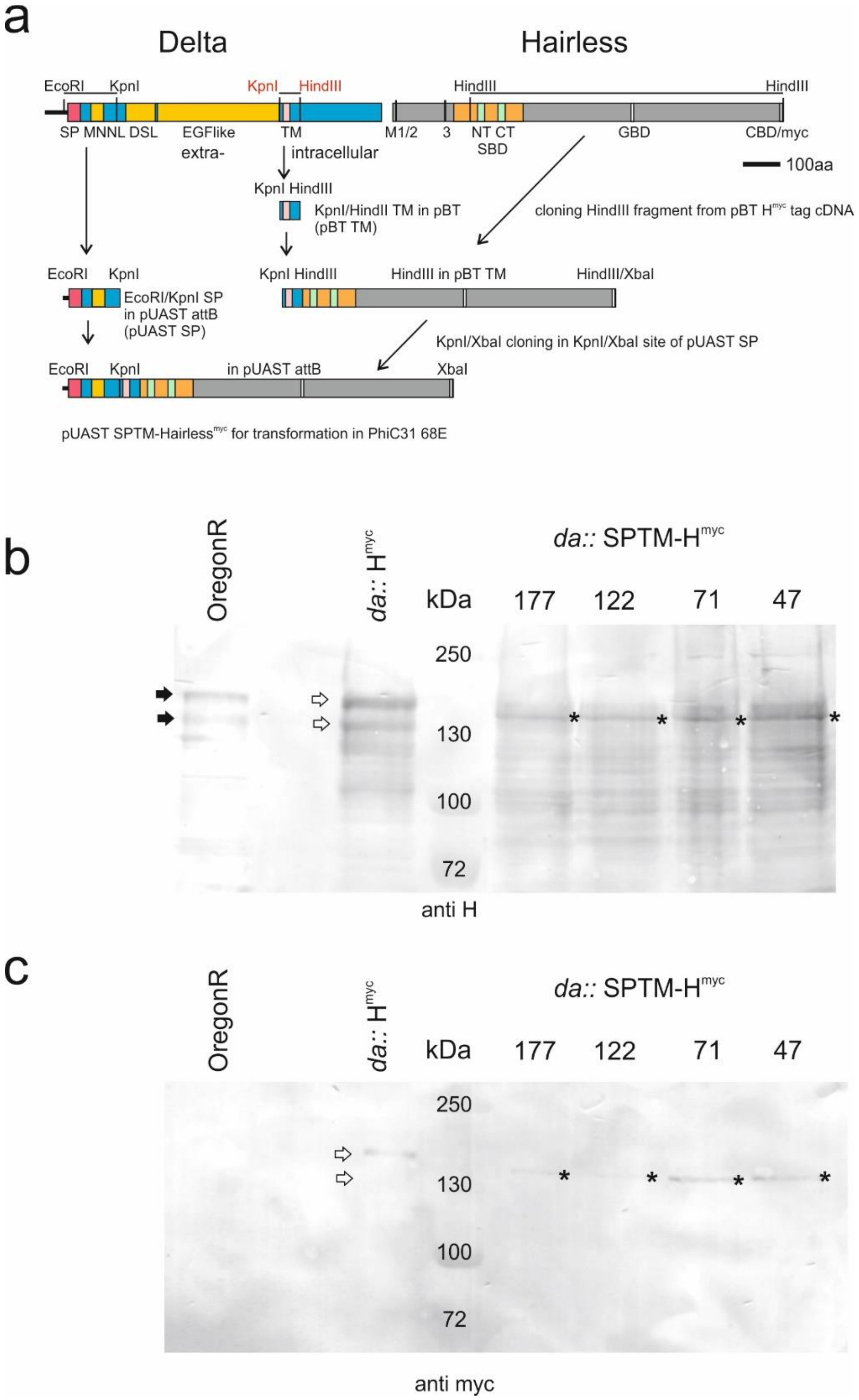

Figure S1: Construction of the *SPTM-H^myc^* transgene. (a) Cloning scheme of the *SPTM-H^myc^* transgene. The Delta signal peptide was subcloned into the pUAST attB vector (pUAST SP). The transmembrane domain was PCR amplified and cloned into the pBluescript (pBT TM) vector. Afterwards Hairless without IRES was cloned into the pBT TM vector, and then into the pUAST SP vector. Restriction sites used for cloning are shown above; red ones were introduced by PCR. SP, signal peptide; MNNL, Notch ligand domain; TM, transmembrane domain; DSL, Delta-Serrate-Lag2 domain; M1/2, regular

translation start in Hairless, M3, IRES-mediated translation start in Hairless, SBD, Suppressor of Hairless binding domain with NT and CT highly conserved sequences [74,75]; GBD, Groucho binding domain; CBD, binding domain of the C-terminal binding protein; myc tag. **(b,c)** Western Blots; approximate size is given in kDa using a prestained marker. Protein extracts derived from Oregon R wild type embryos, and embryos derived from a cross of *da*-Gal4 and either UAS-H^{myc} for control or UAS-SPTM-H^{myc}. Blots were probed with anti-Hairless (b) and anti-myc antibodies (c), respectively. Two protein isoforms of approximately 150 kDa and 120 kDa are derived from the Hairless locus, the smaller one by use of an IRES [42,43] (arrows). The respective calculated molecular weight is 111 kDa and 96 kDa. Membrane-anchored SPTM-H^{myc} protein, however, is detected as a single band of approximately 130 kDa (asterisk) with a calculated molecular weight of 112 kDa (b,c). Specificity was confirmed by detection with anti-Myc antibodies (c).

Figure S2: Sequences of SPTM-H^{myc} and SPTM-GFP

SPTM-H^{myc}

```
MHWIKCLLTAFICFTVIVQVHSSGSFELRLKYFSNDHGRDNEGRCCSGESDGATGKCLGSCKTRFRVCLKHYQAT
IDTTSQCTYGDVITPILGENSVNLTDAQRFQNKGFTNPIQFPFSFSWPGTTNAQVVLIAVFSVAMPLVAVIAACV
VFCMKRKRKRQEKDDAEARKQNEQNAVATSFDMGRTPISTHGNNSWGGYGGRLQFFKDGKFILELARSKDGDKSG
WVSVTRKTFRPPSAATSATVTPTSAVTTAYPKNENSTSLSFSDDNSSIQSSPWQRDQPWKQSRPRRGISKELSLF
FHRPRNSTLGRAALRTAARKRRRPHEPLTTSEDQQPIFATAIKAENGDDTLKAEAAEAVEIENVAVADTTTNEIK
IEKPDTIKGEDDAERLEKEPKKAVSDDSESKEASPGQQVEPQPKDETVDVEMKMNTSEDEEPMTELPRITNAVNG
DLNGDLKASIGKPKSKPKPKAKLSSIIQKLIDSVPARLEQMSKTSAVIASTTTSSDRIGGGLSHALTHKVSPPSS
ATAAGRLVEYHTQHVSPRKRILREFEKVSLEDNGCVNNGSGGASSGGAGGKRSRAKGTSTSSPAGKASPMNLAPP
QGKPSPSPGSSSSSTSPATLSTQPTRLNSSYSIHSLLGGSSGSGSSSFSSSGKKCGDHPAAIISNVHHPQHSMYQ
PSSSSYPRALLTSPKSPDVSGSNGGGGKSPSHTGTKKRSPPYSAGSPVDYGHSFYRDPYAGAGRPSTSGSASQDL
SPPRSSPASPATTPRTVPKKTASIRREFASPSASSSSCPSPGDRSASPPERRHMQQQPHLQRSSPLHYYMYPPPP
QVNGNGSAGSPTSAPPTSNSSAAAVAAAAAAAAAYIPSPSIYNPYISTLAALRHNPLWMHHYQTGASPLLSPHPQ
PGGSAAAAAAAAAARLSPQSAYHAFAYNGVGAAVAAAAAAAAFGQPAPSPHTHPHLAHPHQHPHPAALTTHHSPA
HLATPKLTDSSTDQMSATSSHRTASTSPSSSSASASSSAATSGASSSAMFHTSSLRNEQSSDLPLNLSKHVDMEQ
KLISEEDLE*
```

SPTM-GFP

```
MHWIKCLLTAFICFTVIVQVHSSGSFELRLKYFSNDHGRDNEGRCCSGESDGATGKCLGSCKTRFRVCLKHYQAT
IDTTSQCTYGDVITPILGENSVNLTDAQRFQNKGFTNPIQFPFSFSWPGTTNAQVVLIAVFSVAMPLVAVIAACV
VFCMKRKRKRAQEKDDAEARKQNEQNAVATSLISNSCSPGDPPVATMVSKGILVELDGDVNGHKFSVSGEGEELF
TGEGDATYGKLTLKFICTTGKLVVPPVPWPTLVTTLTYGVQCFSRYPDHMKQHDFFKSAMPEGYVQERTIFFKDD
GNYKTRAEVKFEGDTLVNRIELKGIDFKEDGNILGHKLEYNYNSHNVYIMADKQKNGIKVNFKIRHNIEDGSVQL
ADHYQQNTPIGDGPVLLPDNHYLSTQSALSKDPNEKRDHMVLLEFVTAAGITLGMDELYK*
```

(a) SPTM-H^{myc} sequence. Black Delta with red, signal peptide and light red, transmembrane domain; grey, Hairless; blue, myc tag. Calculated molecular weight SPTM-H^{myc} 111.86 kDa (https://www.bioinformatics.org/sms/prot_mw.html); **(b)** SPTM-GFP sequence. Red, signal peptide; light red, transmembrane domain; light green, sequences from pEGEFP-N1 vector; green, GFP. Calculated molecular weight SPTM GFP 48.46 kDa (https://www.bioinformatics.org/sms/prot_mw.html).

Figure S3: Effect of the combined activity of SPTM-H^{myc} and Su(H) on Wingless expression

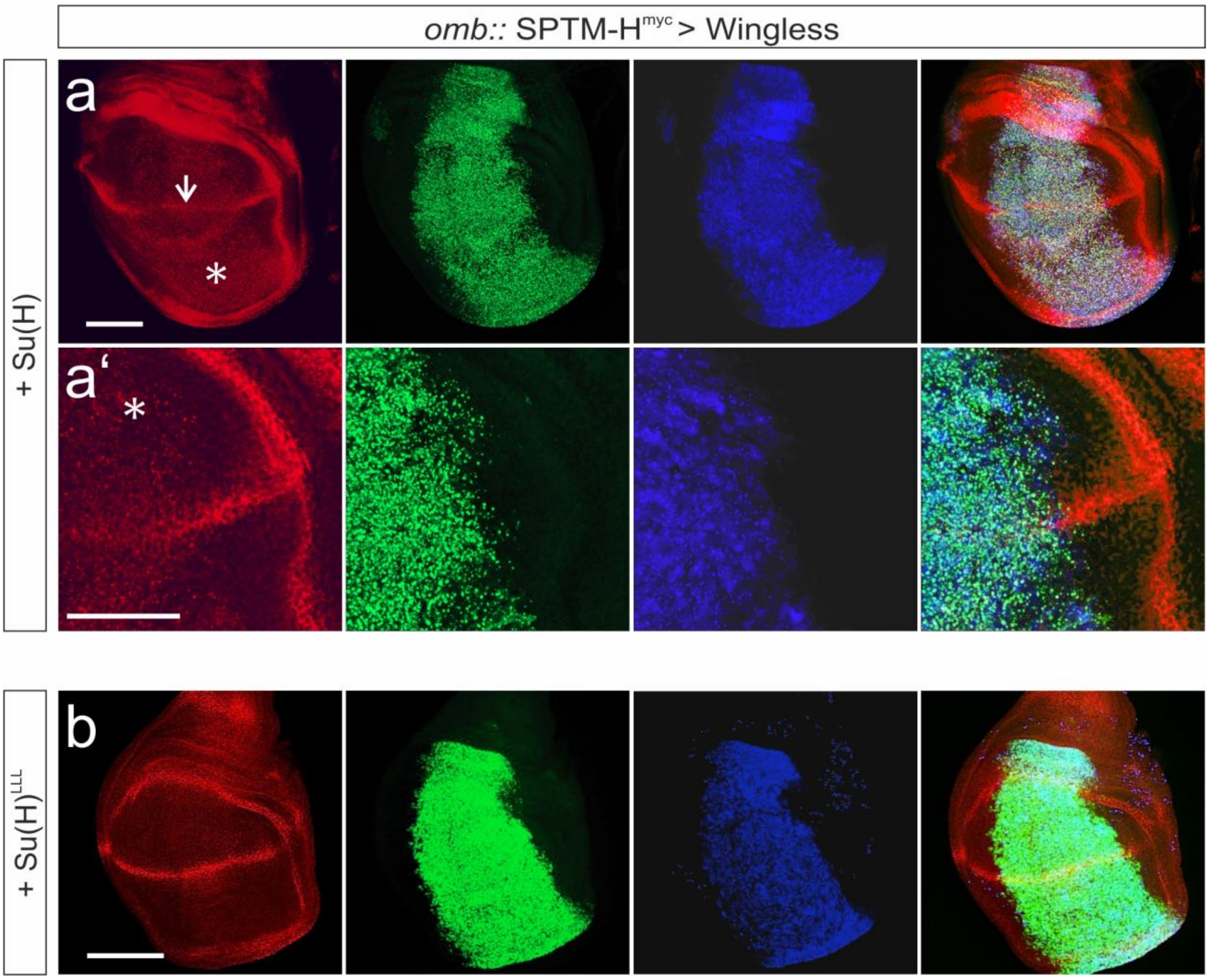

(a,a') *omb*-Gal4 was used to drive co-expression of SPTM-H^{myc} (green) and Su(H) (blue) in the central domain of wing imaginal discs. Wingless (red) expression was downregulated along the dorso-ventral boundary (arrow), but derepressed within the *omb*-domain (asterisk). The disc appeared slightly enlarged. Although less distinct, Su(H) staining follows to a large degree the dotted staining of SPTM-H^{myc} (a'), which appears cyan in the merge; **(b)** Co-induction of SPTM-H^{myc} with Su(H)^{LLL} results in increased disc size, however, Wingless expression was not reduced. Su(H)^{LLL} protein expression was weaker and did not overlap with SPTM-H^{myc} protein. Size bars: 100µm (a,b) and 50µm (a').

Figure S4: Effect of the combined activity of SPTM-H^{myc} and Su(H) on Cut expression

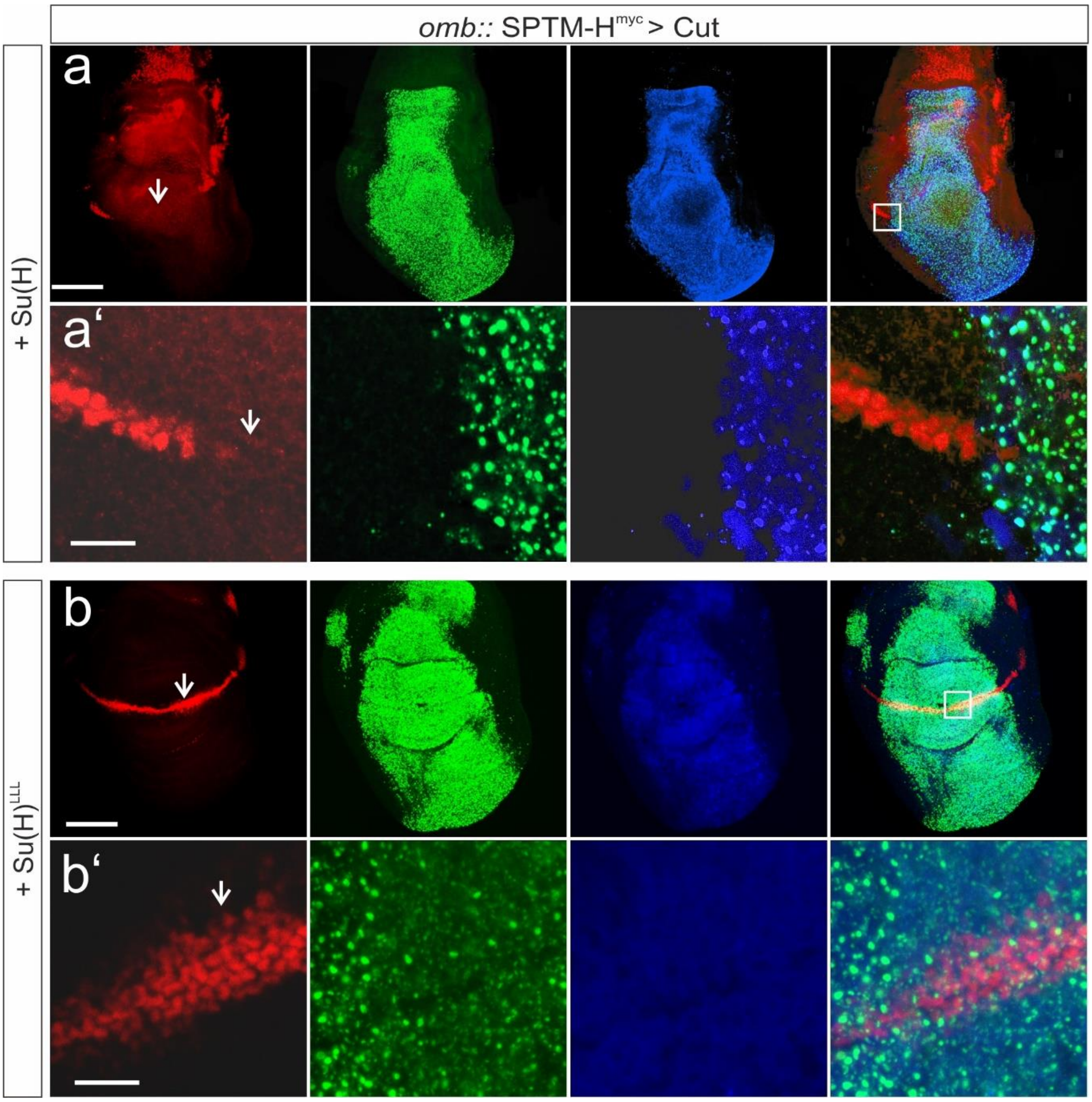

(a,a′) SPTM-H^{myc} (green) and Su(H) (blue) were co-expressed in the central domain of wing imaginal discs using the *omb*-Gal4 driver. Cut (red) expression was repressed along the dorso-ventral boundary (arrow), and expanded elsewhere within the *omb*-domain. The disc appeared slightly enlarged. Vesicular co-localization of Su(H) and of SPTM-H^{myc} (a′) is apparent in the merge (cyan color), albeit Su(H) protein accumulation appears more uniform; **(b,b′)** The combined overexpression of SPTM-H^{myc} (green) and Su(H)^{LLL} (blue) caused enlarged discs, without impeding Cut (red) expression along the boundary or inducing it outside (arrow). The Cut expression domain, however, comprised more than the normal 2-3 rows of nuclei (b′, compare with a′). Mutant Su(H)^{LLL} protein accumulated uniformly at a remarkably low level, and did not overlap with SPTM-H^{myc}. Size bars: 100µm (a,b); 50µm (a′,b′).

Figure S5: Effects on the size of the *omb*-expression domain

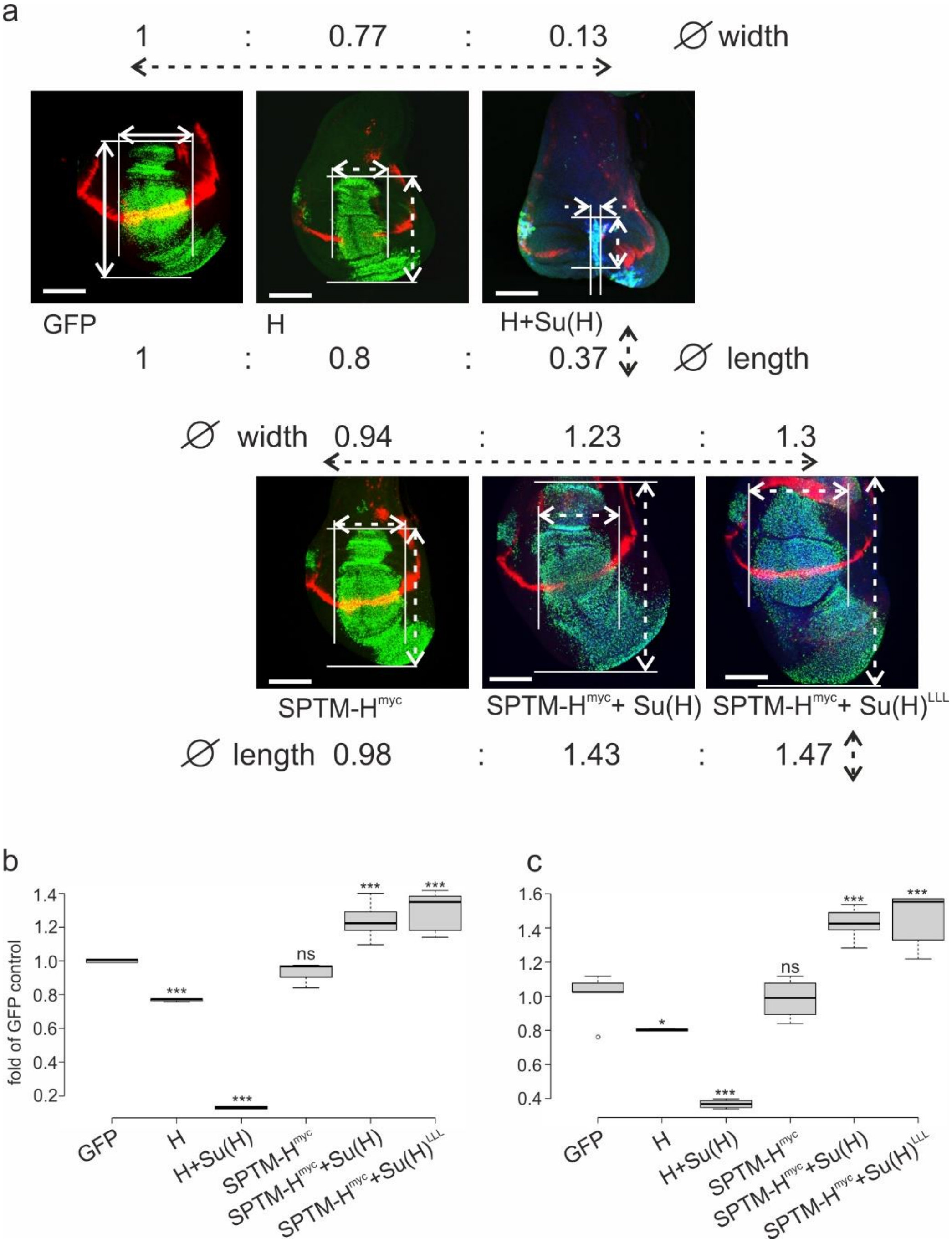

Overexpression within the *omb*-domain of the given constructs. **(a)** Pictures were taken from Figure 5 (upper row) and Figure 7 (lower row) to demonstrate size measurements. The width and length of the *omb* expression domain was recorded from several specimens; the average number relative to GFP-control is shown above and below, respectively. For width measurements, vg^{BE} expression was used as lead. Box blot representation of the statistical analysis on width **(b)** and length **(c)** measurements. Center lines show medians, box limits indicate 25th and 75th percentiles as determined by R software; whiskers extend 1.5 times the interquartile range from the 25th and 75th percentiles; outliers are represented by dots. n= 5, 4, 4, 4, 14, 6 sample points. ANOVA two-tailed Dunnet's test for multiple comparisons was applied relative to GFP-control (*** <0.001; * <0.01; ns >0.05).
